# Recent Progress of Powering IoT Based on Thermoelectric Technology

**DOI:** 10.3390/mi16091017

**Published:** 2025-08-31

**Authors:** Jinhong Dai, Haitao Deng, Jingwen Huang, Xiaosheng Zhang

**Affiliations:** 1 School of Integrated Circuit Science and Engineering, University of Electronic Science and Technology of China, Chengdu 611731, China; thomasdai1126@outlook.com (J.D.); jingwen_huang2024@163.com (J.H.); 2 Institute of Industrial Science, The University of Tokyo, Tokyo 153-8505, Japan

**Keywords:** MEMS, thermoelectric generator, seeback effect, IoT

## Abstract

With the rapid advancement of electronic devices, Internet of Things (IoT) technology has become increasingly integrated into everyday life. However, its broader development has been restricted by challenges related to long-term maintenance and the frequent need for power source replacements. Among the available power supply solutions, thermoelectric power generation has garnered significant interest due to its high reliability. Nevertheless, the widespread application of thermoelectric generators (TEGs) in IoT remains limited due to their relatively low conversion efficiency and structural fragility. This review systematically summarizes recent strategies aimed at enhancing the output performance and durability of TEGs through improvements in manufacturing processes and performance optimization techniques. It highlights several fabrication methods capable of endowing devices with superior flexibility and reliability, including screen printing, chemical vapor deposition (CVD), and electrospray deposition. Additionally, we discuss two key approaches for improving power generation performance: advanced material selection and multi-mechanism hybridization. Finally, the article explores the applications of TEGs in thermal energy harvesting from wearable devices, ambient environments, and aerospace fields, demonstrating their substantial potential to provide sustainable energy for IoT devices.

## 1. Introduction

Since the concept of the Internet of Things (IoT) was introduced in 1999, the IoT market has been expanding at an unprecedented rate [1,2], playing a big role in our daily lives. With the continuous increase in the number of IoT nodes, their energy consumption is also rising rapidly [3,4,5]. Consequently, additional energy sources are required to power these nodes. In recent years, various methods for electricity generation, including wind, hydro, and solar energy [6,7,8,9,10,11,12], have been developed. However, not all power supply methods can adequately meet the complex demands of the IoT, particularly when portability and stability are essential. Additionally, the maintenance of IoT devices can be costly, and regular battery replacement often leads to substantial resource waste. Furthermore, pollution remains a critical concern [13,14]. A considerable amount of energy is wasted in daily life, making the harvesting and conversion of such energy into usable power a significant challenge [15,16,17]. Given these considerations, thermoelectric energy harvesting technology emerges as a promising and reliable source of electricity among various power generation methods [18,19].

**Figure 1:**
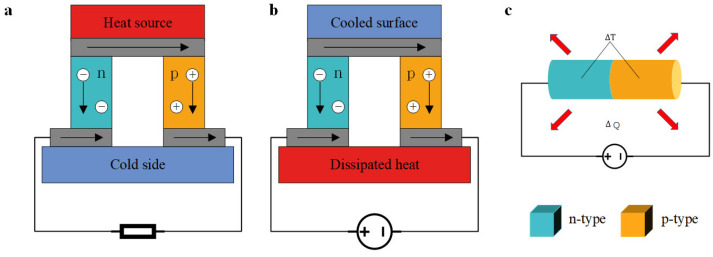
Three power generation principles of thermoelectric generators. (**a**) Seebeck effect. (**b**) Peltier effect. (**c**) Thomson effect.

The mutual conversion of thermal energy and electrical energy was discovered over two centuries ago, including the Seebeck effect, the Peltier effect, and the Thomson effect. Their fundamental principles are illustrated in Figure 1. When a temperature gradient exists between the two ends of a TEG, charge carriers migrate in a uniform direction. When p-type and n-type thermocouples are arranged and connected end-to-end, a voltage, referred to as the Seebeck voltage, can be generated between the hot and cold terminals, as depicted in Figure 1a. 

When a current flows through two distinct materials, heat is absorbed at one end and released at the other, a phenomenon known as the Peltier effect, as illustrated in Figure 1b. Additionally, when a temperature gradient is applied to a uniform conductor carrying a current, further heat absorption or release occurs, referred to as the Thomson effect, as depicted in Figure 1c.

Among them, TEGs generate electricity mainly through the Seebeck effect. The Seebeck voltage can be expressed as:

(1) V=α×T

where V represent the Seebeck voltage, *α* represents the Seebeck coefficient, T represents the absolute temperature.

Thermoelectric performance is not measured by the size of the Seebeck voltage, but by the thermoelectric figure of merit (ZT):

(2) $$\mathrm{ZT}=\frac{\alpha^{2}\sigma T}{\kappa }$$

where *α* represents the Seebeck coefficient, T represents the absolute temperature, *κ* represents the thermal conductivity, and σ represents the electrical conductivity. *κ* can be divided into *κ_e_* and *κ_l_*. *κ_e_* represents electron thermal conductivity, *κ_l_* represents lattice thermal conductivity.

**Figure 2:**
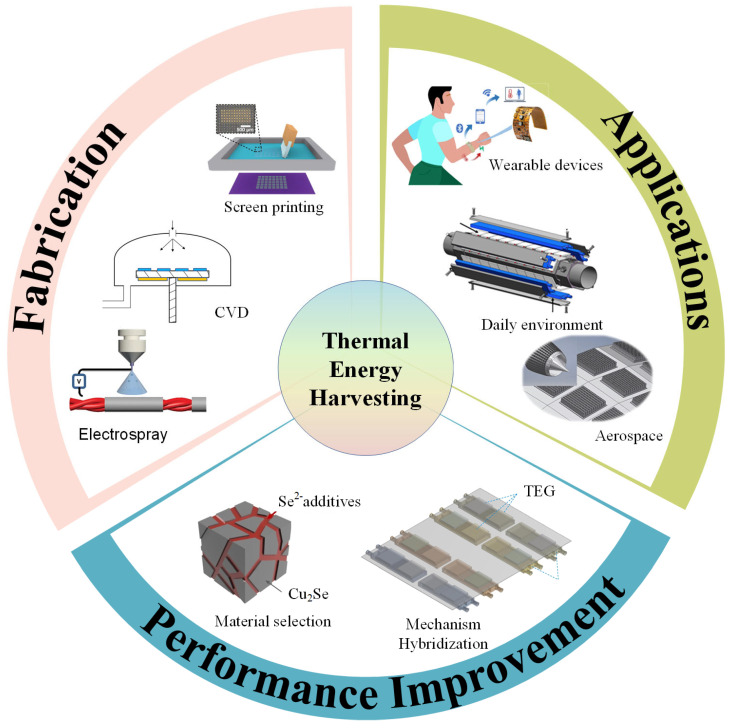
Overview of recent progress of powering IoT based on TEG, including fabrication method (i.e., screen printing, chemical vapor deposition (CVD), electrospray, reprinted with permission from Ref. [20]. 2023, Nature.), performance improvement (i.e., material selection, reprinted with permission from Ref. [21]. 2024, Nature. Hybrid generator, reprinted with permission from Ref. [22]. 2023, Elsevier.), application (wearable device, reprinted with permission from Ref. [23]. 2024, Wiley-VCH Verlag. Daily environment, reprinted with permission from Ref. [24]. 2023, Elsevier., Aerospace, reprinted with permission from Ref. [25]. 2023, SAGE.).

As shown in Figure 2, we overview the current progress of powering IoTs based on thermoelectric technology. The technical meaning can be summarized as follows: (1) We overviewed the development of TEGs from bulk-, film-, to yarn-shaped TEGs, and figured out the suitable materials and fabrication methods for realizing each shape of TEGs with good durability. (2) Two effective strategies to improve thermoelectric outputs are emphasized, including high-performance thermoelectric materials and mechanism hybridization with other power generation principles, such as photovoltaic, electrostatic, and piezoelectric effects. (3) Practical IoT applications of the bulk-, film-, and yarn-shaped TEGs in wearable devices, daily environments, and the military industry (such as aerospace) were summarized. Challenges and feasible technique solutions of future thermoelectric systems for sustainable powering the IoTs were discussed.

## 2. Fabrication Method of TEG

**Figure 3:**
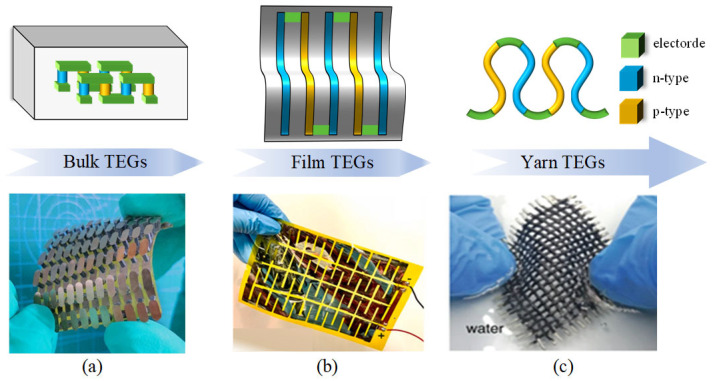
Structure configuration of TEGs (**a**) Bulk-shaped. Reproduced with permission from Elsevier (2020). Reprinted with permission from Ref. [31]. 2023, Nature. (**b**) Film-shaped. Reprinted with permission from Ref. [32]. 2021, Elsevier. (**c**) Yarn-shaped. Reprinted with permission from Ref. [33]. 2020, Springer Nature.

As illustrated in Figure 3, TEGs can be classified into three primary categories: bulk, film, and yarn. Since the 1950s, bismuth telluride has served as a fundamental thermoelectric material for the fabrication of bulk TEGs [26]. By the 1970s, manufacturing processes had progressively matured, resulting in the development of smaller and lighter bulk TEGs. Concurrently, there has been a growing focus on flexible TEGs. With advancements in micro-electromechanical systems after the year 2000, film- and yarn-based TEGs were introduced [27]. A significant number of thermoelectric materials have been developed and implemented in practical applications. These improvements have markedly enhanced the portability and reliability of TEGs, facilitating their use as power sources for increasingly compact Internet of Things (IoT) devices [28,29,30]. Consequently, an increasing number of fabrication methods are now being adopted in industrial and commercial production. In the following section, we provide an overview of several commonly used manufacturing techniques.

### 2.1. Sintering

Sintering is a traditional method initially employed for the fabrication of bulk TEGs [34,35,36]. However, due to the high costs and irreversible effects on materials during high-temperature processes, this technique has gradually phased out. Recent advancements have focused on enhancing the sintering method through various approaches, including plasma spark sintering, cold sintering, and pre-sintering.

In contrast to conventional sintering, Spark Plasma Sintering (SPS) can significantly reduce the sintering temperature, processing time, and the adverse effects of high temperatures on materials. For instance, F. Giovannelli et al. successfully synthesized La_7_Mo_7_O_30_ [37] using the SPS process, which required only 10 min and resulted in ceramics with exceptionally low thermal conductivity, thereby greatly improving the efficiency of thermoelectric power generation.

The elevated temperatures associated with traditional sintering techniques can compromise the micro/nanostructure of thermoelectric materials and introduce chemical issues. Piyawat et al. demonstrated the fabrication of a dense sample at a temperature of 473 K using Cold Sintering Process (CSP) [38], achieving a density comparable to samples produced by hot pressing at higher temperatures. This method significantly reduces thermal conductivity, primarily through the inhibition of lattice thermal conductivity (*κ_l_*), which enhances phonon scattering at grain boundaries due to restricted grain growth. The resulting ZT value reached 2.13, which is notably high compared to existing thermoelectric materials.

### 2.2. Screen Printing

**Figure 4:**
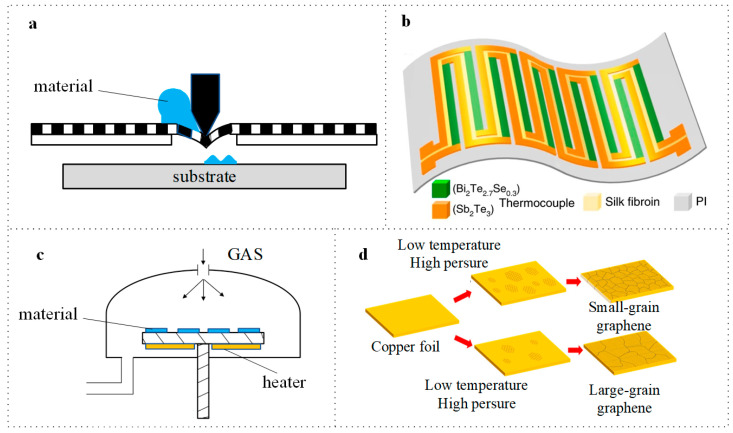
(**a**) Schematic of screen print process. (**b**) A flexible and reliable TEG prepared by screen printing. Reprinted with permission from Ref. [42]. 2020, Nature. (**c**) Schematic of chemical vapor deposition (CVD) process. (**d**) Synthesizing graphene with smaller grain size by CVD. Reprinted with permission from Ref. [45]. 2018, MDPI.

Screen printing is a widely utilized technique for producing film-shaped items [39,40,41]. The working principle is illustrated in Figure 4a. These TEGs exhibit significantly low production costs and demonstrate considerable adaptability to various flexible environments. For instance, Wen et al. printed Bi_2_Te_2.7_Se_0.3_ (n-type) and Sb_2_Te_3_ (p-type) thermoelectric inks on a flexible polyimide (PI) substrate using screen printing, as depicted in Figure 4b. This method facilitates hybrid sensing functionality without complex preparation processes [42]. Additionally, devices fabricated through screen printing are highly suitable for large-scale manufacturing and commercialization. Zhang et al. successfully produced flexible Ag_2_Se/terpineol composite films via screen printing [43]. Furthermore, researchers are enhancing traditional screen-printing techniques to accommodate a broader range of materials. Liu et al. successfully printed TEGs [44] of In_2_O_3_ and ITO on polyimide substrates by refining traditional screen-printing methods. The TEG demonstrated an exceptionally high Seebeck coefficient of 175.8 μV/°C and maintained excellent operational stability even at temperatures of 199 °C. 

### 2.3. Chemical Vapor Deposition

Due to the simple operation and easy access to raw materials, CVD is one of the primary fabrication methods for film preparation [46,47,48]. The working principle is illustrated in Figure 4c. CVD is widely utilized in the manufacturing of thermoelectric devices and can be employed to prepare various thermoelectric materials, including chalcogenides, tellurides, and oxides. The interfaces significantly influence the performance of thermoelectric devices, and CVD enables precise control over the interface properties between thin films and substrates, thus optimizing device performance.

Through CVD, we can prepare thermoelectric devices with nanoscale structures such as nanowires and nanoparticles. These nanostructures can enhance the thermoelectric properties of materials, improving both the efficiency and stability of devices. Recent advancements in traditional CVD methods have been made to obtain better thermoelectric characteristics. For example, Gyumin Lim et al. synthesized graphene using the CVD method [45], achieving a smaller grain size at low temperature and high pressure, which resulted in a higher nucleation density, as depicted in Figure 4d. The resultant graphene exhibits increased density and improved ZT characteristics. Additionally, Rositawati et al. incorporated gold to form an ohmic junction during the preparation of multilayer graphene via CVD [49]. This adjustment of carrier concentration subsequently increased the ZT value.

### 2.4. 3D Printing

**Figure 5:**
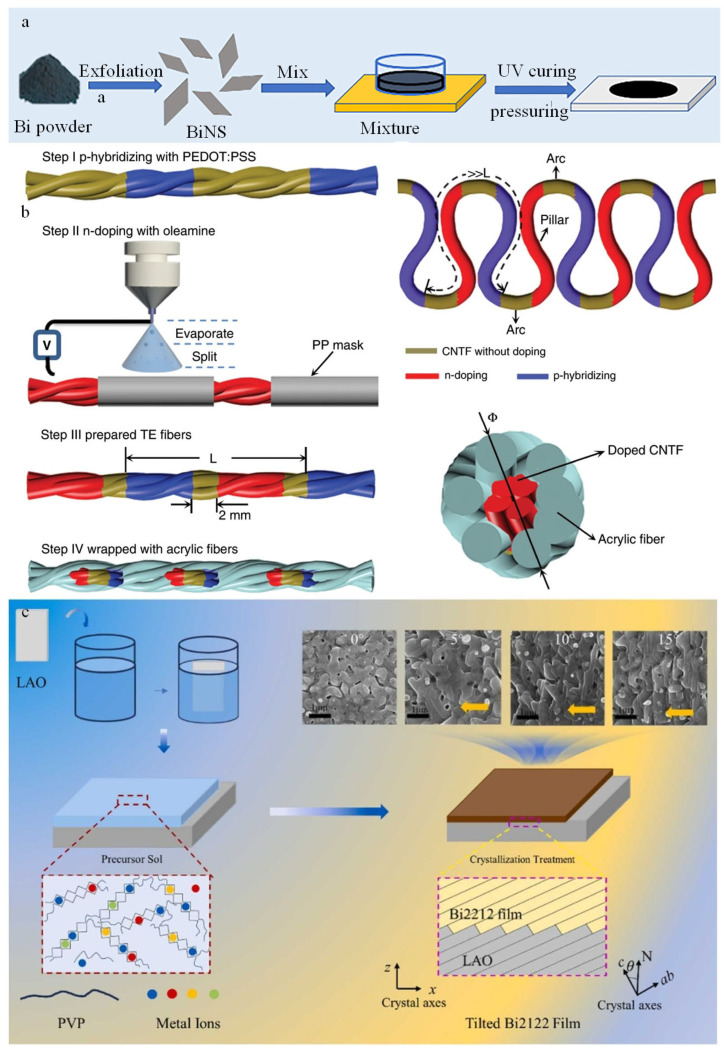
(**a**) TEG made out of 3D printing. Reprinted with permission from Ref. [52]. 2024, John Wiley and Sons. (**b**) Fabrics using electrospray technology to harvest thermo energy. Reprinted with permission from Ref. [20]. 2020, Springer Nature. (**c**) TEG based on Sol-gel method. Reprinted with permission from Ref. [53]. 2024, Elsevier.

3D printing technology is an advanced manufacturing process that enables users to create three-dimensional objects by layering materials [50]. This technology has a wide range of applications, including the production of customized products, bioprinting in the medical field, and construction within the construction industry. TEGs can also be produced by 3D printing techniques [51]. As illustrated in Figure 5a, Meng et al. utilized bismuth nanosheets (BiNS) to fabricate thermoelectric devices through UV-curing 3D printing [52]. While the Seebeck coefficient, ZT, does not show a significant increase, 3D printing facilitates the creation of more complex shapes.

### 2.5. Electrospray Technology

Other fabrication methods for thermoelectric devices have limitations in terms of shape. Electrospray technology offers a viable solution for producing thermoelectric fabrics. As illustrated in Figure 5b, Sun et al. utilized electrospraying to fabricate a carbon-nanotube-based TEGs [20]. In the initial step depicted in Figure 5b-step (i), the process begins with immersing carbon nanotube fibers (CNTF) into a commercial poly(3,4-ethylenedioxythiophene): poly(styrenesulfonate) (PEDOT: PSS) solution. A polypropylene mask (PP) is then applied to the substrate, followed by n-type hybridization through electrospray and oleamine doping, as shown in Figure 5b-step (ii). Subsequently, thermoelectric legs with alternating doping are formed, as indicated in Figure 5b-step (iii). To prevent short circuits, the doped CNTF is encapsulated in acrylic fibers using coverspinning technology, resulting in the desired TEG, with an output of 70 mW·m^−2^ at 44 K and notable bending resistance.

### 2.6. Sol-Gel Method

The sol-gel process is a unique method for the qualitative and quantitative synthesis of high-purity metal oxide nanomaterials characterized by ultra-fine structures and single-phase particles [54,55,56]. Zhao et al. utilized this sol-gel methodology to fabricate Bi2212 thin films on miscut single-crystal LaAlO_3_ substrates [53]. The resulting films displayed anisotropic properties, rendering them appropriate for applications in thermoelectric devices, as illustrated in Figure 5c.

The manufacturing process of TEG is continuously evolving, with a focus on increased flexibility and miniaturization. However, this evolution does not imply the abandonment of previous methodologies. For large-scale waste heat power generation applications, the low-cost and high-efficiency sintering route remains the preferred approach. In contrast, film printing technology is poised to lead in flexible manufacturing and micro-device applications. Additionally, specialized manufacturing processes such as combination of Nanosynthesis and SPS will be required for extreme conditions. Concurrently, cost-effective, large-scale laboratory advancements will emerge, promoting the large-scale commercial application of thermoelectric technology.

## 3. Performance Improvement

In addition to structural problems, the performance of TEGs needs to be improved to ensure a higher output. We introduce the optimization of the TEG in two ways: material selection and hybrid power generation.

### 3.1. Material Selection

Among all thermoelectric materials, bismuth telluride [57,58,59] remains the most widely utilized. Its ZT value is approximately 1 at 50 °C and decreases at higher temperatures. The manufacturing process for bismuth telluride has reached a mature stage.

**Figure 6:**
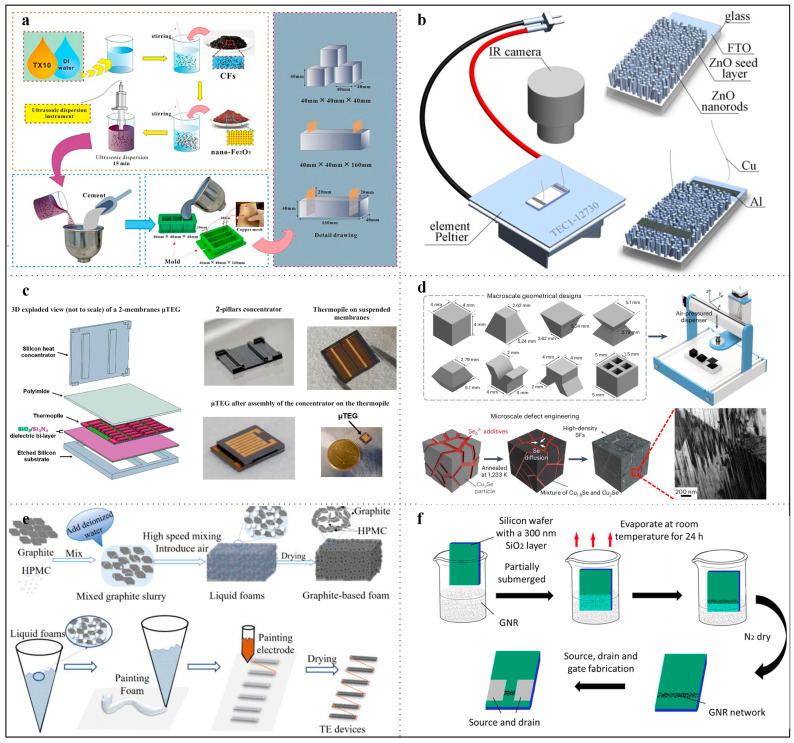
Thermoelectric materials for TEG. (**a**) Nano Fe_2_O_3_/carbon fiber (CF)/cement composite. Reprinted with permission from Ref. [60]. 2023, Elsevier. (**b**) TEG based on ZnO/FTO. Reprinted with permission from Ref. [61]. 2019, Elsevier. (**c**) TEG made out of Cu_55_Ni_45_ and Ni_90_Cr_10_. Reprinted with permission from Ref. [53]. 2024, Springer Nature. (**d**) TEG made out of Cu_2_Se. Reprinted with permission from Ref. [21]. 2024, Springer Nature. (**e**) TEG based on graphite. Reprinted with permission from Ref. [63]. 2023, AIP. (**f**) Graphene based TEG. Reprinted with permission from Ref. [64]. 2023, Elsevier.

Since the discovery of the Seebeck effect, researchers have actively investigated many thermoelectric materials. Recently, there has been a growing focus on various metal oxides, metal compounds, and novel materials. For example, Wan et al. developed a nano Fe_2_O_3_/carbon fiber (CF)/cement composite [60]. Figure 6a illustrates the fabrication process. The composite of nano Fe_2_O_3_, carbon fiber (CF), and polyoxyethylene nonyl phenyl ether (TX10) disperser in the cement matrix endowed the cement composite with a high Seebeck coefficient (1.1234 mV/K), as well as good compressive strength and flexural strength. This demonstrates its practical application on the surfaces of urban buildings and sidewalks for urban energy harvesting. As depicted in Figure 6b, Klochko et al. developed a translucent solar thermoelectric generator based on ZnO/FTO [61]. Although this device achieves a V_oc_ of 0.28 mV, a short-circuit current of 220 nA, and a maximum output power of 13 pW, it is capable of integration with glass windows to capture near-infrared and ambient heat from outdoor sunlight.

In addition to metal oxides, metal compounds exhibit superior thermoelectric performance. As shown in Figure 6c, Bel-Hadj et al. fabricated a thermocouple using Cu_55_Ni_45_ and Ni_90_Cr_10_. This metallic material effectively reduces the internal resistance of the TEG, achieving an output of 125 μW/cm^2^ [62]. Cu_2_Se is an efficient thermoelectric material that has garnered extensive attention due to its excellent thermoelectric properties, low toxicity, and abundant elemental reserves. As demonstrated in Figure 6d, Choo et al. prepared Cu_2_Se using a 3D printing method, a low-temperature process that preserves the microstructure of Cu_2_Se, resulting in a higher ZT value [21]. 

Besides metals, graphite and graphene [49,65] demonstrate excellent electrical and thermal conductivity. Graphite functions as a p-type semiconductor and exhibits n-type semiconductor behavior when combined with polyethylenimine (PEI). For instance, as illustrated in Figure 6e, Duan et al. developed a graphite-based foam through a mechanical foaming process [63]. The foam samples underwent compression treatment to transform the three-dimensional foam into a porous film, thereby enhancing power generation efficiency. As depicted in Figure 6f, Wei et al. fabricated a graphene nanoribbon (GNR)-based TEG [64]. The edge effect of GNR can reduce carrier concentration, which is inversely related to the Seebeck coefficient. Consequently, increasing the bandgap can enhance the Seebeck coefficient. The experimental results show a Seebeck coefficient of 6.76 μW·m^−1^K^−2^, which is significantly higher than that of traditional graphene-based TEGs. Furthermore, Table 1 provides a list of additional materials.

The introduction highlights that traditional thermoelectric materials like bismuth and tellurium, can achieve favorable output at room temperature. However, their high cost is driven by the scarcity of rare elements, the cost is extremely high, and the rare elements are harmful to the human body and cause pollution. Metal oxides are capable of operating at elevated temperatures, offering a cost-effective and non-toxic alternative. Nonetheless, they are challenged by low ZT values and fragile structures. While metal mixtures exhibit high performance and low thermal conductivity, their preparation methods are quite complex. It’s difficult to manufacture and commercialize on a large scale. Conductive polymers, although inexpensive and flexible, fall short in output capacity and do not meet the necessary stability requirements.

### 3.2. Mechanism Hybridization

**Figure 7:**
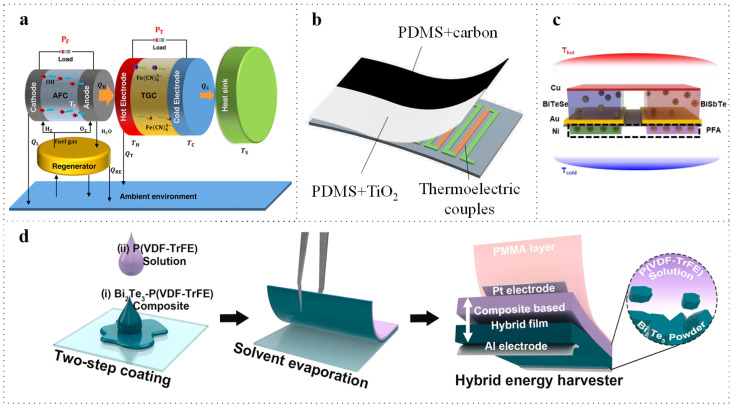
Hybrid generator. (a) Fuel cells combined with TEG. Reprinted with permission from Ref. [71]. 2024, Elsevier. (b) TEG hybrid with a light-to-thermal conversion layer to simultaneously harvest thermal and radiation energies based on a single working mechanism. Reprinted with permission from Ref. [72]. 2021, ACS. (c) A triboelectric and thermoelectric hybrid generator. Reprinted with permission from Ref. [73]. 2023, Elsevier. (d) A piezoelectric and thermoelectric hybrid generator. Reprinted with permission from Ref. [74]. 2023, Elsevier.

To enhance output performance, TEGs can be integrated with other generators to improve power generation efficiency. Fuel cells can generate electricity and heat without moving parts, typically exhibiting high efficiency and low emissions; however, they also produce a significant amount of waste heat. The integration of TEGs with fuel cells can improve power generation efficiency. In Figure 7a, Wang et al. designed a hybrid power system [71] consisting of an alkaline fuel cell (AFC) module, a heat accumulator, a TEG unit, and a heat sink. This system enables the TEG to capture the waste heat generated by the AFC and convert it into electricity. The maximum power density of the hybrid system is 105.71% of that of the stand-alone AFC system. Triboelectric generators represent a less mature technology that can similarly be combined with TEGs. The photovoltaic thermal (PVT) system has emerged as a promising direction in recent years. The combination of a TEG with a photovoltaic generator can cool the PV panels and generate electricity while collecting waste heat from the PV panels. There are ongoing improvements to existing PVT systems. In Figure 7b, Wen et al. present a flexible hybrid photo-thermoelectric generator (PTEG) featuring a simple structure composed of a TEG and a light-to-thermal conversion layer, enabling simultaneous harvesting of thermal and radiation energies based on a single working mechanism [72]. By incorporating light-absorbing and reflective layers, the power generation efficiency is significantly enhanced. As illustrated in Figure 7c, Kim et al. achieved an output increase of 1.75 times by combining triboelectric and thermoelectric generators [73]. In Figure 7d, Kim et al. integrated a TEG with a piezoelectric generator (PEG) to power a wearable human sensing network [74]. This thermoelectric-piezoelectric hybrid generator (TPHG) was entirely monolithic and fabricated using a straightforward droplet-casting method. This power generation approach enables simultaneous energy harvesting from multiple sources without being limited to a specific condition. Additionally, the device demonstrated improved stability over 5000 cycles of durability testing.

Hybrid power generation has demonstrated its effectiveness in enhancing energy conversion efficiency. In the future, it will serve as a fundamental solution for diversifying power sources. This approach addresses the limitations of relying on a single power generation environment and facilitate a more stable energy supply. Although challenges such as microintegration, material compatibility, and cost management still exist, advancements in experimental research will lead to the emergence of more hybrid generators. This evolution will contribute to a new era of interconnected intelligent systems, fostering energy self-sufficiency.

## 4. Application of TEG in IoT

Due to the small size and good reliability, TEG is widely used in IoT. Based on energy consumption, we will introduce the application of TEGs in the following categories: wearable devices, waste heat recovery, and aerospace applications. In this section, we summarize these thermoelectric energy harvesting devices in detail.

### 4.1. Wearable Applications

Wearable electronics often require stable and continuous energy sources [75]. The human body is a constant source of heat. As mentioned in Chapter 1, most thermoelectric devices today are film-shaped using a planar process. Therefore, TEGs are suitable for wearable devices. 

**Figure 8:**
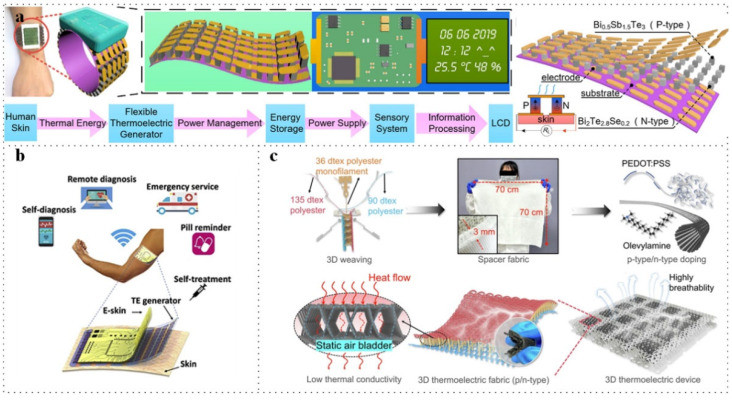
Wearable devices based on TEG in IoT. (**a**) Bulk-TEG made as watch. Reprinted with permission from Ref. [76]. 2020, Elsevier. (**b**) Film-TEG powered E-skin. Reprinted with permission from Ref. [77]. 2019, Elsevier. (**c**) Yarn-TEG-made cloth generator. Reprinted with permission from Ref. [78]. 2025, Springer Nature.

As illustrated in Figure 8a, Yuan et al. developed a highly efficient flexible thermoelectric generator (f-TEG) utilizing bismuth telluride grains assembled on a flexible polyimide substrate [76]. The self-powered bracelet is capable of continuously monitoring temperature, humidity, and acceleration, which can be further processed to calculate human stride frequency and analyze gait in motion. These measurements are processed in the microcontroller unit (MCU) and displayed in real time on an LCD. In Figure 8b, Tian et al. introduced a concept for a film-based thermoelectric generator (film-TEG) integrated with electronic skin [77]. Electronic skin holds significant market potential, with an estimated £1.7 billion market for wireless health monitoring systems alone. The TEG can be utilized to generate electricity, which can then power these electronic skins through power management circuits and high-efficiency energy storage units. Numerous studies on film-TEG have already been conducted. In Figure 8c, He et al. fabricated a three-dimensional flexible thermoelectric device featuring an inner rigid and outer flexible woven design [78]. This device has the potential for integration into everyday wearable garments; in this work, it was successfully incorporated into an N95 mask to monitor human breathing.

Despite these advantages, TEGs are not yet widely deployed at scale. This limitation arises from the inability of current TEG materials to simultaneously meet the demands for flexibility and output power. Additionally, the integration of the complete power generation system with power consumption equipment presents significant challenges. To date, simultaneous miniaturization and integration of TEG technology remain unachieved. These challenges will constitute the primary research directions for wearable TEG systems in the future.

### 4.2. Daily Environment Applications

In addition to the human body serving as a heat source, significant waste heat is generated in everyday life. Examples include automobile exhaust [79], road waste heat [80] and industrial waste heat [81]. These heat sources can be harnessed, and TEGs can be utilized to power Internet of Things (IoT) devices.

**Figure 9:**
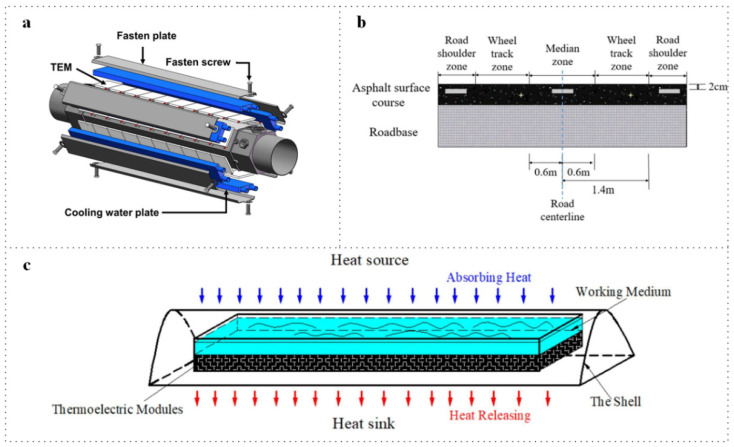
Waste heat recovery application. (**a**) exhausted heat recovery. Reprinted with permission from Ref. [24]. 2023, MDPI. (**b**) road wasted heat recovery. Reprinted with permission from Ref. [82]. 2022, Springer Nature. (**c**) daily heater with TEG to recover waste heat. Reprinted with permission from Ref. [84]. 2023, Elsevier.

As illustrated in Figure 9a, this model features an automotive TEG equipped with hot and cold ends, thermoelectric modules (TEMs), and fastening structures [24]. The TEMs are bismuth-telluride-based devices arranged in a Π shape, comprising 126 pairs of thermoelectric legs. Sixty TEMs were assembled onto six hot surfaces, with ten TEMs allocated per surface. Li et al. proposed a genetic algorithm-back propagation (GA-BP) model based on this design, which enhances the accuracy of the TEG and provides both experimental and theoretical foundations for TEG design and numerical simulations. In Figure 9b, Xie et al. explored the installation of a thermoelectric module on a road surface [82]. This module absorbs heat from asphalt pavements while mitigating the urban heat island effect [83]. Their study analyzes the factors influencing thermoelectric generator utilization (TEGU) in pavement power generation, including burial depth, pavement defects, and the number of series modules. Temperature differentials are not only present outdoors but can also be harnessed indoors. 

In Figure 9c, Zhang et al. fabricated a waste heat recovery device aimed at reducing the energy consumption of an electric water heater [84]. This device consists of a shell, a thermoelectric unit, and a working medium. The working medium captures heat from the heat source to create an artificial low-temperature zone. Compared to traditional heaters, the enhanced water heater effectively absorbs waste heat from the top of the unit. In addition to heat sources influenced by human activity, numerous natural heat sources exist, with geothermal energy being one of them. The hot springs in Thailand maintain high temperatures year-round, presenting an opportunity for energy regeneration. Mona et al. deployed a thermoelectric module under the base of a hot spring to leverage geothermal energy for powering an IoT detection unit [85]. This setup monitors various parameters, including air temperature, air humidity, water temperature, cooling water temperature, sulfur dioxide (SO_2_) levels, current, voltage, and the output of the TEG module.

TEG is designed to work for a long time once deployed, making it friendly for IoT devices that need to be maintained. But TEG is still not widely used on waste heat recovery, because the corresponding technology is not mature enough. Compared to batteries, TEG is not stable enough and requires additional power management circuits and energy storage components, which is a large work. After miniaturizing the entire system, there will be more IoT nodes in the future, and TEG can power these micro sensor nodes at low cost.

### 4.3. Aerospace Application

**Figure 10:**
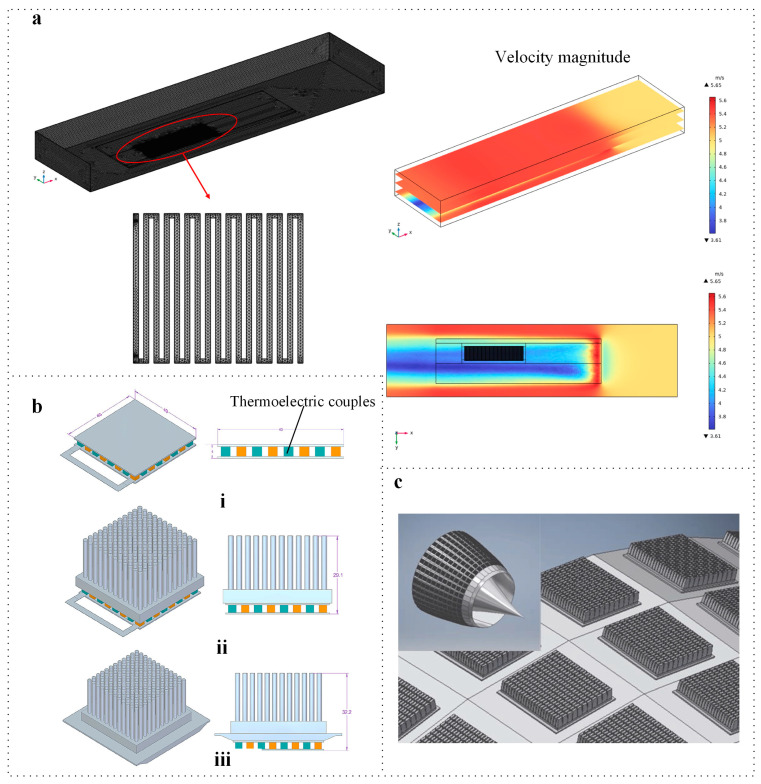
TEG applications in aerospace. (**a**) TEGs applied to plane engine. Reprinted with permission from Ref. [86]. 2024, Elsevier. (**b**) A schematic diagram of the TEG on an engine: (**i**) a standalone TEG; (**ii**) a TEG with a heat sink alone; (**iii**) A TEG integrated with a heat sink and vapor chamber. Reprinted with permission from Ref. [87]. 2018, MDPI. (**c**) TEG with vapor chamber in aerospace. Reprinted with permission from Ref. [25]. 2021, SAGE.

In the process of aerospace, it is inevitable to use thrusters with large energy consumption. A considerable amount of waste heat is generated during thruster operation, which is a significant source of energy. If harnessed effectively, this energy can lead to reduced energy consumption and minimized environmental pollution for aircraft. A survey conducted by Boeing indicated that TEGs could decrease fuel consumption by 0.5%, which translates into operating cost savings of tens of millions of dollars. Consequently, numerous researchers have dedicated their efforts to related studies. For example, Doragi et al. examined the performance of TEGs based on polymer nanocomposites during various flight phases [86]. They utilized a model, as illustrated in Figure 10a, where the cold end temperature ranged from −15 °C to 1 °C, and the hot end ranged from 63 °C to 41 °C. The output voltage varied between 67 mV and 116 mV, with measurements from simulation softwares showing significant consistency, indicating that TEGs in aerospace can be evaluated and simulated more effectively. In Figure 10b, Ziolkowski et al. explored the application of a TEG for jet engine nozzles through finite element modeling [87]. 

During thermal power generation, heat is continuously transferred to the cold end. Therefore, it is essential to consider the heat dissipation at this end. As illustrated in Figure 10c, Sarris et al. employed a vapor chamber coupled with a conventional circular needle-fin heat sink to enhance the output power of TEGs, effectively reducing the temperature of the cold junction [25]. A TEG integrated with a heat sink and vapor chamber demonstrates superior heat dissipation compared to a standalone TEG (Figure 10c(i)) or a TEG with a heat sink alone (Figure 10c(ii)). However, further research is necessary to explore the application of this method in aircraft engines, particularly under dynamic air conditions.

During space exploration, a significant portion of energy is inaccessible, and in extreme environments, even basic lighting cannot be guaranteed. Consequently, thermal energy is utilized for electricity generation. Radioisotope thermoelectric generators (RTGs) were first deployed in space for electricity generation in the 1950s. These generators do not rely on nuclear fusion or fission; instead, they harness the natural radioactive decay of plutonium-238, primarily in the form of plutonium dioxide (238PuO_2_), for thermal power generation. RTGs are characterized by low quality yet high reliability, enabling them to operate for years or even decades. They have been successfully utilized in missions such as Voyager 1 and Voyager 2, providing energy as they journey to the edge of the solar system. Ongoing optimizations are being made to RTGs; for instance, the Multi-Mission Radioisotope Thermoelectric Generator (MMTRG) incorporates multiple nested layers, allowing it to function effectively in both atmospheric and vacuum conditions. Researchers are actively investigating the performance of RTGs in space. For example, Whiting et al. conducted a study examining the lifetime and performance of MMTRGs and analyzed their power variation behavior during exploration [88].

The extremely high lifespan makes RTGs capable of many space missions. But the lack of radioelement becomes a big problem. That’s why RTG is not widely used today. Another issue is the power output. RTGs can only provide a few hundred watts of power. For greater energy demands, we can only rely on better nuclear reactors.

## 5. Conclusions and Prospect

In recent years, researchers made efforts on developing thermoelectric technologies and reported meaningful research papers and reviews. There are many entry points to summarize thermoelectric generators (TEGs) and its applications for the fields of IoTs. For TEGs, the entry points include but not limited to thermoelectric materials (traditional, flexible or stretchable materials) [89,90,91], fabrication of TEGs in different shapes (bulk-, film-, and yarn-shapes) and how these structures affect the performance of TEGs in outputs, weight, user conformability and integrity, etc. [92,93,94], performance evaluation of the reported TEGs and discussion of how these key factors (materials, structures, and mechanism) affect TEG outputs and practical applications [95,96]. For IoT applications of TEGs, the entry points involve sustainable energy supplying and self-powered sensing [97,98]. Particularly in sustainable energy supplying, many related technologies are worthy of in-depth discussion, such as power management method for effectively storing and supplying to IoT components, construction of multifunctional TEG system for IoT powering and sensing, and AI-assisted TEG systems. 

Differently from others, in this review, we focus on the current challenges of TEGs in device durability and output enhancement, summarize the recent development of TEGs from bulk-, film-, to yarn-shaped TEGs, figured out the suitable materials and fabrication methods for realizing each shape of TEGs with good durability, and emphasize two effective strategies (material selection and mechanism hybridization) to improve thermoelectric outputs. Finally, we summarized the practical IoT applications of the bulk-, film-, and yarn-shaped TEGs in wearable devices, daily environments, and the military industry (such as aerospace).

In the future, we believe more researches will focus on the development of TEGs with higher efficiency, more flexible applications, more intelligence system with AI technique, and multi-technology hybrid generation. In practice, further development of the fabrication methods and materials is required. Advanced manufacturing techniques lead to more reliable thermoelectric performance. New materials offer higher ZT values and lower thermal conductivity. Noticeably, organic thermoelectric materials are also emerging, adapting to various flexible conditions. Moreover, some environmental-friendly materials are taking place of the traditional thermoelectric material. The integration of TEG systems should improve to minimize. These factors are effective in improving TEG performance. Thus, playing a more important role in IoT.

In general, thermoelectric power generation devices for the IoT is an attractive direction. Attractive potential in wearable electronics, waste heat recovery, aerospace, etc. A dream of Battery-free IoT is in sight. As predicted by “Thermoelectric Generator Market Size & Share Report, 2030” from GRAND VIEW RESEARCH, the global thermoelectric generator market size was estimated at USD 813.38 million in 2023 and is projected to reach $1610.05 million by 2030, growing at a compound annual growth rate of 10.5% from 2024 to 2030. Undoubtedly, many IoT devices will take thermoelectric power generation as one uniform power supply in the future, which has great commercial potential, even though most TEGs are currently in the experimental stage and difficult to fully commercialize.

## Figures and Tables

**Table 1:** Summary of different thermoelectric materials in thermoelectric performance.

Composition	Temp. (°C)	|*α*| (μV·K^−1^)	ZT	Ref.
Bi_2_Te_3_	340	120	1.1	[26]
Bi_2_Te_2.7_Se_0.3_	370	180	1.0	[66]
CNTs		60		[67]
Ag_2_Se/PVP		140		[68]
PEDOT:PSS/SnSe		100		[69]
Skutterudites	845		1.45	[70]

## Data Availability

No new data were created or analyzed in this study.
